# Food Supply of the Child

**Published:** 1914-12-15

**Authors:** Walter H. Jackson

**Affiliations:** Ann Arbor


### FOOD SUPPLY OF THE CHILD.

BY WALTER H. JACKSON, D.D.S., ANN ARBOR.

In looking over the many things that occur in a life experience, nothing seems of more importance than the food supply for the child.

That the food supply be so balanced, that all the tissues of the body shall be nourished and perfectly developed so they may perform the function for which they were created.

Each organ requires certain food constituents for its development and should these constituents be lacking, there comes a call to supply that deficiency. A hunger that is not satisfied by eating, and which, if continued, produces a weary or tired feeling, and the child cannot concentrate his mind on his studies, becomes careless about everything that interests children.

The man that labors must have food to supply the wear and tear of the muscles, and when it is withheld, he also, will soon tire. So with the child that is growing. Each part must have its necessary supply of pabulum, or there will be a weariness, which if long continued, may dwarf the mind of the child to such an extent that it will be always infantile in its development from not being able to use the mental faculties to develop them. Many young growing children in the public schools seem to be listless and tired. They do not get their lessons, not being able to concentrate their minds, to the aggravation of their teachers. Often the child is not to blame in the least.

Teachers are irritated and may be discouraged when they are unable to secure satisfactory results with such children. To illustrate by cases: Mr. M.— brought to me his son, 14 years of age, five feet eight inches or over, in height, but a baby in mind, as he could not pass the third grade. His teacher said, "I do not want him to come back to my room again." The father had employed physicians for him since he was six years old hoping something could be done to help him, but all to no purpose. Finally Dr. G— referred him to me to see if spreading the Maxillae might not be of benefit.

On examination I found the six and twelve year molar crowns cut down to the gums, but no pulps exposed. The Maxillae needed to be expanded in order to make room for bringing his teeth into position, but what would produce this condition of the teeth? I asked him how he felt, his reply, "I'm so tired all the time, I cant think," in a whining way like a baby.

I told the father that the boy was starving and that no child could study or apply his mind to anything while in that condition. I prescribed calcium phosphate daily and asked him to report to me in three days. The third day he returned as directed, and on inquiry as to how he felt, he said, "I feel bully, my tired feeling is all gone." He further said that he did not think he ever felt so well before in his life. I advised the father to procure a person to coach him in his studies for the summer, which was done. The boy made good and entered the fourth grade a changed boy.

The same conditions may occur during gestation. I give one out of many cases: Mrs. C.— called me to examine her teeth as to their condition. She complained of feeling tired all the time and her physician was unable to help her, and she asked me what to do. I told her it was hunger that made her tired, and prescribed phosphate of lime or phosphate of soda daily. She came back two days later to have some filling done. All her tired feeling was gone and she said she could walk a mile. The need of an extra measure of tissue building material to supply the growing child she was carrying, made its demand on the mother, hence the hunger.

Other cases of both mothers and children might be given, but further narration is unnecessary. As a rule I prefer plain calcium phosphate and let the digestive organs by their own solvents take up that which the system needs. It is the food that they need, not the medicine.

